# Supplementary material for: Dynamic metastable long-living droplets formed by sticker-spacer proteins
Source: eLife. 2020 Jun 2;9:e56159. doi: 10.7554/eLife.56159 (PMC7360371; doi:10.7554/eLife.56159)
Supplement: Transparent reporting form [file elife-56159-transrepform.docx]

***eLife’s* transparent reporting form**

We encourage authors to provide detailed information *within their submission* to facilitate the interpretation and replication of experiments. Authors can upload supporting documentation to indicate the use of appropriate reporting guidelines for health-related research (see [EQUATOR Network](http://www.equator-network.org/  \t _blank)), life science research (see the [BioSharing Information Resource](https://biosharing.org/)), or the [ARRIVE guidelines](http://www.plosbiology.org/article/info:doi/10.1371/journal.pbio.1000412) for reporting work involving animal research. Where applicable, authors should refer to any relevant reporting standards documents in this form.

If you have any questions, please consult our Journal Policies and/or contact us: [editorial@elifesciences.org](mailto:editorial@elifesciences.org).

**Sample-size estimation**

- You should state whether an appropriate sample size was computed when the study was being designed
- You should state the statistical method of sample size computation and any required assumptions
- If no explicit power analysis was used, you should describe how you decided what sample (replicate) size (number) to use

Please outline where this information can be found within the submission (e.g., sections or figure legends), or explain why this information doesn’t apply to your submission:

**Replicates**

This is a coarse grained simulation study to measure the dynamics of cluster growth and its dependence on physical quantities of the polymer. The concept of sample size does not apply to this study. However, we provide a detailed rationale for choice of model parameters and their values in the Methods section of the article.

- You should report how often each experiment was performed
- You should include a definition of biological versus technical replication
- The data obtained should be provided and sufficient information should be provided to indicate the number of independent biological and/or technical replicates
- If you encountered any outliers, you should describe how these were handled
- Criteria for exclusion/inclusion of data should be clearly stated
- High-throughput sequence data should be uploaded before submission, with a private link for reviewers provided (these are available from both GEO and ArrayExpress)

Please outline where this information can be found within the submission (e.g., sections or figure legends), or explain why this information doesn’t apply to your submission:

Our study used multiple independent LD and MC simulations to compute first moments of raw, measurable quantities such as cluster sizes and molecular exchange times (mentioned in Statistical Reporting below). We do not present findings from any experiments in this article.

**Statistical reporting**

- Statistical analysis methods should be described and justified
- Raw data should be presented in figures whenever informative to do so (typically when N per group is less than 10)
- For each experiment, you should identify the statistical tests used, exact values of N, definitions of center, methods of multiple test correction, and dispersion and precision measures (e.g., mean, median, SD, SEM, confidence intervals; and, for the major substantive results, a measure of effect size (e.g., Pearson's r, Cohen's d)
- Report exact p-values wherever possible alongside the summary statistics and 95% confidence intervals. These should be reported for all key questions and not only when the p-value is less than 0.05.

Please outline where this information can be found within the submission (e.g., sections or figure legends), or explain why this information doesn’t apply to your submission:

(For large datasets, or papers with a very large number of statistical tests, you may upload a single table file with tests, Ns, etc., with reference to sections in the manuscript.)

**Langevin Dynamics Study:**

Fig.2, Fig.5, Fig.9 -- The histograms are directly plotted by calculating first moments of single largest cluster sizes, or raw cluster size distributions. The quantities were computed for 500 different configurations (from the last 1 microsecond of a 16 microsecond simulation run) across 5 independent trajectories. Fig 4. The mean first passage times were computed using 100 independent dimerisation simulations.

**Monte Carlo Study:**

All the mean cluster sizes in the MC simulations were computed over 100 independent kinetic Monte carlo trajectories. The mean molecular exchange times were computed over a 100 independent trajectories of 10 hour long simulation runs.

**Group allocation**

- Indicate how samples were allocated into experimental groups (in the case of clinical studies, please specify allocation to treatment method); if randomization was used, please also state if restricted randomization was applied
- Indicate if masking was used during group allocation, data collection and/or data analysis

Please outline where this information can be found within the submission (e.g., sections or figure legends), or explain why this information doesn’t apply to your submission:

**Additional data files (“source data”)**

This study employs coarse-grained simulations and hence there is no

group allocation of samples.

- We encourage you to upload relevant additional data files, such as numerical data that are represented as a graph in a figure, or as a summary table
- Where provided, these should be in the most useful format, and they can be uploaded as “Source data” files linked to a main figure or table
- Include model definition files including the full list of parameters used
- Include code used for data analysis (e.g., R, MatLab)
- Avoid stating that data files are “available upon request”

Please indicate the figures or tables for which source data files have been provided:

All of our figures are based on raw 1-D or 2-D histograms of the raw data. In the Model and the Methods sections of our article, we outline the values of physical parameters used in our simulations as well as the rationale behind these values. We also include the source data for all the Figures in the form of a compressed zip file containing the raw data files. Also, part of the compressed zip file is the LAMMPS simulation script as well as a representative LAMMPS data file.
